# Problems in accessing healthcare among women in India: a district-level change analysis, 2016–2021

**DOI:** 10.1186/s12889-026-26392-7

**Published:** 2026-02-06

**Authors:** Shalem Balla, Rockli Kim, Sujata Saunik, S. V. Subramanian

**Affiliations:** 1 School of Humanities and Social Sciences (SHSS), Indian Institute of Technology, Mandi, Himachal Pradesh India; 2 Division of Health Policy and Management, College of Health Science, Korea University, Seoul, South Korea; 3 Interdisciplinary Program in Precision Public Health, Department of Public Health Sciences, Graduate School of Korea University, Seoul, South Korea; 4 Independent researcher, Mumbai, Maharashtra India; 5 Harvard Center for Population and Development Studies, Boston, MA USA; 6 Department of Social and Behavioral Sciences, Harvard T. H. Chan School of Public Health, Boston, MA USA

**Keywords:** Problems in accessing healthcare, Women's health, Affordability, Proximity, Permission, Support

## Abstract

**Background:**

Limited access to healthcare services among women leads to adverse health outcomes such as high maternal mortality, untreated chronic conditions and poor reproductive and child health.

**Methods:**

We used data from India’s National Family Health Survey 2015–16 and 2019–21 to examine district-level changes in the percentage of women facing problems in accessing healthcare (Affordability, Proximity, Permission and Support/Companionship). We also estimate how much improvement has occurred in the districts for each outcome variable. Using a geospatial method, we aligned the district geometries between two survey rounds to strengthen the policy relevance of this research. We also conducted a supplementary analysis by rural and urban areas.

**Findings:**

Overall, the percentage of women experiencing problems in accessing healthcare has declined from 2016 to 2021. The most significant reduction was observed in proximity barrier, which declined from 63.20% to 56.79%. Affordability as a problem has declined over the period, but still remains as a major barrier in eastern and Northeastern India. Rural women consistently face more problems in accessing healthcare than urban women, with proximity and affordability being major problems. Overall, the percentage of women who reported all four problems has reduced from 27.15% (95% CI: 27.05 – 27.26) in 2016 to 23.19% (95% CI: 23.09—23.29) in 2021.

**Interpretation:**

The observed variation in barriers to accessing healthcare by women at the district level underscores the need for a decentralized approach in healthcare policy-making with strategies focused on district-specific challenges and priorities.

**Supplementary Information:**

The online version contains supplementary material available at 10.1186/s12889-026-26392-7.

## Introduction

Access to healthcare is a basic human right and a critical determinant of health. Lack of healthcare access, especially for women of reproductive age may lead to adverse health outcomes such as high maternal mortality, untreated chronic conditions and poor reproductive and child health [1–3]. However, healthcare access remains a critical challenge for many people across the globe [4, 5]. A systematic study conducted by Kowal and colleagues [6] show that unmet need for healthcare access among 60 + population ranges from less than 2% in countries like Andorra, Qatar, Republic of Korea, Slovenia, Thailand and Viet Nam to over 50% in Georgia, Haiti, Morocco, Rwanda, and Zimbabwe [6]. In India alone, 12% of respondents reported not using healthcare despite a perceived need.

Healthcare access is mostly discussed in terms of supply-side factors such as financial barriers or availability of healthcare facilities; which have led to the expansion of demand-side financing policies or healthcare facilities. For instance, in India, the Ayushman Bharat scheme was launched in 2017 that aimed to provide cashless coverage to the bottom 40% of the population, and simultaneously increase primary healthcare access through wider service coverage and more facilities [7]. Despite such efforts, in 2021 India, 62% of people reported an unmet need for treatment in public hospitals and this percentage was significantly higher among women and children of socioeconomically disadvantaged groups [8–10].

This raises the question if there are barriers beyond traditional supply-side factors like distance to health facility, availability of medicines and doctors and financial hardship that influence the ability of patients to access healthcare [11]. Evidence shows that 34.2% of women reported facing problems in getting permission to go to healthcare facilities, 56.0% reported problems in taking transport, 49.1% reported problems with going alone to healthcare facilities, and 61.6% of women reported problems with the unavailability of female healthcare providers [12, 13]. With all the above supply and demand side factors combined, nearly 84.0% of females of reproductive age experienced some problems in accessing health care (PAHC) in India in 2019 [14, 15]. Thus, PAHC among women of reproductive ages in India are multi-faceted and context-specific, and range from supply-side barriers such as distance to healthcare facilities, and cost of treatment to issues of travelling alone or acquiring permission to go to health facilities [16, 17]. These factors can present themselves in combination or isolation, however their context may change across geographic regions. For instance, while permission to go to health facilities may be a problem for females due to empowerment or safety concerns in northern states, in the northeast it may be an outcome of geographical difficulties [18–20].

So far in India, the existing literature has focused on objective indicators of PAHC. Yet a growing body of evidence highlights that subjective perceptions are also important in determining healthcare access behaviour [21, 22]. Women may report distance, transport, affordability and permission as major problems, even though the services are technically available and these reflects issues such as poor transport facilities, safety concerns and cultural norms. These subjective perceptions are powerful determinants of healthcare seeking and utilisation. By utilizing these self-reported measures of PAHC, our study captures the lived reality and provides more in-depth understanding of how structural and perceptual factors jointly influence healthcare-seeking behaviour.

Further, evidence suggests that demographic, socio-cultural factors, physical, political, economic and gender-based factors are affecting the access to healthcare [23–26]. This evidence underscores the geographic variation and questions the applicability of uniform macro-level policies to address these issues effectively. Demographic and sociocultural factors vary significantly across the districts, as do the implementation and impact of healthcare policies [27]. Moreover, physical conditions and infrastructural disparities contribute to the unequal distribution of healthcare challenges. These regional differences highlight the limitation of state-level or national-level averages which often obscure critical within-state inequalities.

Therefore district-level analysis is essential to capture the nuanced nature of healthcare access barriers in India. This is crucial for designing targeted interventions that address the unique needs of a particular region. Moreover, the district-level analysis enables policymakers to allocate the resources more efficiently, prioritizing high-burden regions and implementing tailored solutions that have a higher chance of success than the one-size-fits-all approach.

Despite the gravity of these issues, there are limited studies that simultaneously quantify the problems using self-reported indicators at the district-level, explore how these problems vary across geography and track changes over time. By moving beyond simple counts, our analysis offers new insights into the experience of problems by women and where the policy efforts should be prioritised. To facilitate such policy dialogues, we provide district-level estimates. Further, to ensure comparability across 2016 and 2021 clusters were redistributed to align with current political boundaries, resulting in a dataset encompassing 720 districts relevant to the current administrative framework.

## Methods

### Data and sampling strategy

This analysis utilized data from the fourth and fifth rounds of India’s National Family Health Survey (NFHS) conducted in 2015–2016 and 2019–2021, respectively. From this point forward, we will refer exclusively to the end year of each survey for simplicity. Both surveys are components of the Demographic and Health Surveys Program (DHS) and collect data about population health, nutrition, and well-being. Within the entire scope, the surveys include indicators related to barriers to accessing healthcare. Both surveys were explicitly designed to provide estimates nationally, state-level and district-level and structured to identify clusters—rural villages and urban wards—using probability proportionate to size from districts within states. Subsequently, households were randomly selected from each cluster. The latest NFHS (2021) report contains a comprehensive overview of the sampling strategy utilized [14].

### Study population

The 2021 dataset contains information from 724,115 women aged 15–49 years belonging to 30,161 clusters across 36 states and Union Territories (UTs). Information on PAHC was collected from all women aged 15–49 years. Since there are no missing values in the indicators of interest, we have included all 724,115 women from 2021. The 2016 dataset contains information from 699,689 women aged 15–49 years belonging to 28,467 clusters across all 36 states and UTs. However, we excluded 1,310 women due to the reorganization of clusters to align with the districts of 2021. Consequently, the total number of women included in 2016 was 698,379. Since the present study does not explore any socio-economic variation; we do not consider any missing values other than for our outcome variables.

### Outcome

We examined four problems of accessing healthcare as outcomes of this study. These were: getting the money needed for treatment, distance to a health facility, getting permission to go, and not wanting to go alone. From this point forward we use the terms ‘Affordability’, ‘Proximity’, ‘Permission’ and ‘Support/Companionship’ to represent these problems, respectively. Each question had three response options: ‘No problem’, ‘Not a big problem’ and ‘Big problem’. For the analysis, we created a binary variable for each of these four outcomes, whereby 'Any problem' includes both ‘Not a big problem’ and ‘Big problem’.

### District geometry

This study aims to analyze district-level variations in PAHC by women across India and how these changed between 2016 and 2021. Due to administrative updates, the number of districts in India has increased from 640 in 2016 to 707 in 2021. To ensure comparability, we use a 2022 configuration of 720 districts. This updated district configuration is used in place of the 707 districts because the state of Andhra Pradesh (AP) created 13 new districts in April 2022 [28]. The inclusion of these new districts is crucial, as none of the 2021 districts from AP aligns with the updated district boundaries, rendering any meaningful interpretation of PAHC within the state impossible without this adjustment. The underlying methodology to update the district boundaries is described in detail elsewhere [29].

### Statistical analysis

The NFHS data are organized with women (i) nested within clusters (j), districts (k), and states (l). Utilizing this nested structure, we employed a Markov Chain Monte Carlo (MCMC) based multilevel modeling to estimate the prevalence of the problems of Affordability, Proximity, Permission, and Support/Companionship across each district in India for the years 2016 and 2021. MCMC methods adhere to a Bayesian framework wherein prior information is utilized to optimize a likelihood function as given below: [30, 31]

$$logit \left({P}_{ijkl}\right)= {\beta }_{0}+\left({C}_{0jkl}+ {D}_{0kl}+ {S}_{0l}\right)$$

For each of the four outcomes in each survey round, ${\beta }_{0}$ represents the constant, and ${C}_{0jkl} , {D}_{0kl}$, and ${S}_{0l}$ are the residual differentials for clusters j, districts k, and states l, respectively. The computed priors were subsequently employed to implement MCMC on the identical model. We designated a burn-in period of 500 cycles and monitored 5,000 iterations of chains [30, 32]. This combination enabled us to determine an effective sample size (ESS) for each model (Supplementary Table 1). The estimates of residual differentials were obtained using the *runmlwin *command in Stata 18 [33]. The MCMC residuals were then used to calculate the precision-weighted estimates for the share of each outcome in each cluster:

$$\mathrm{exp}{[\beta }_{0}+({C}_{0jkl}+ {D}_{0kl}+ {S}_{0l})] /[1+\mathrm{exp}{[\beta }_{0}+({C}_{0jkl}+ {D}_{0kl}+ {S}_{0l})]$$

Subsequently, we averaged the cluster estimates for each district.

We utilized the district averages from 2016 to establish decile thresholds for each outcome indicator. These thresholds were employed for the 2021 district averages to illustrate the variation in the proportion of each outcome over time for each district. We additionally computed the disparity between the district-level averages for each outcome in 2016 and 2021. We utilized absolute increase/decrease values to establish five categories of temporal change: Very Low Improvement (< 5.0), Low Improvement (5.0–10.0), Moderate Improvement (10.0–15.0), High Improvement (15.0–20.0), and Very High Improvement (> 20.0).

## Results

### Sample characteristics

The study is based on unit records of 1,422,494 women aged 15 to 49 years of which 724,115 records are from 2021 and 698,379 from 2016 (Table 1). In both years, the largest share of women reported having affordability and proximity problems in accessing healthcare (Table 2). The percentage of women who reported not having a problem for each of the four outcomes was comparatively higher in urban areas than rural for both 2016 and 2021 (Supplementary Tables 2 and 3).

**Table 1:** Study sample size selection from the two National Family Health Surveys, 2016–2021

Questions related to Problems in Accessing Health Care (PAHC)	Sample size based on inclusion criteria (n)	Observations dropped due to re-arrangement of clusters	Final Study Sample size (n)
**NFHS-5 (2019–21)**			
*Getting the money needed for treatment is a problem (Affordability)*	7,24,115	0	7,24,115
*Distance to a health facility is a problem (Proximity)*	7,24,115		7,24,115
*Getting permission to go hospital is a problem (Permission)*	7,24,115		7,24,115
*Not wanting to go alone is a problem (Support/Companionship)*	7,24,115		7,24,115
**NFHS-4 (2015–16)**			
*Getting the money needed for treatment is a problem (Affordability)*	6,99,689	1,310	6,98,379
*Distance to a health facility is a problem (Proximity)*	6,99,689		6,98,379
*Getting permission to go hospital is a problem (Permission)*	6,99,689		6,98,379
*Not wanting to go alone is a problem (Support/Companionship)*	6,99,689		6,98,379
**All waves**	**14,23,804**	**1,310**	**14,22,494**

Bold values represent the "overall" or "pooled"

**Table 2:** Sample size (N) and weighted percentage of women aged 15–49 years who reported Problems in Accessing Healthcare (PAHC) in India, 2016–2021

Variables	2021		2016	
	N	%	N	%
**Affordability**				
*No problem*	3,40,163	49.42	3,13,684	45.25
*Any problem*	3,83,952	50.57	3,84,695	54.75
**Proximity**				
*No problem*	2,86,900	43.22	2,44,239	36.80
*Any problem*	4,37,215	56.79	4,54,140	63.20
**Permission**				
*No problem*	4,67,751	65.28	4,30,530	59.89
*Any problem*	2,56,364	34.73	2,67,849	40.12
**Support/Companionship**				
*No problem*	3,56,159	51.84	3,30,525	48.62
*Any problem*	3,67,956	48.17	3,67,854	51.38

Any problem includes both 'big problem' and 'not a big problem'

### All India trends

In 2016, 45.25% of females aged 15 to 49 years reported having no issue with affordability, while 54.75% identified affordability as a problem (Table 2). By 2021, the percentage of women reporting affordability as a problem decreased to 50.57%. Proximity was identified as a problem by 63.20% of women in 2016, which declined to 56.79% in 2021. Regarding permission, 40.12% of women reported it as a problem in 2016, which fell to 34.73% by 2021. Support/Companionship was considered a problem by 51.38% of women in 2016, declining to 48.17% by 2021. The pattern across different types of problems remains similar to national pattern in rural and urban areas (Supplementary Tables 2 and 3).

### Combination of different problems in accessing healthcare

In 2016, 27.15% (95% CI: 27.05–27.26) of women reported experiencing all four problems and this percentage reduced to 23.19% (95% CI: 23.08–23.29) in 2021 (Table 3**)**. The percentage of women facing any three problems out of four has reduced from 16.80% (95% CI: 16.71—16.89) in 2016 to 15.55% (95% CI: 15.47—15.63). The women who reported the combination of affordability & proximity & support/companionship had the highest share in both 2016 and 2021.

**Table 3:** Percentage of women aged 15–49 years reported different types of problems in accessing healthcare (PAHC) in all possible combinations in India

**Combination of Problems**	Any problem			
	2021	95% Confidence Interval	2016	95% Confidence Interval
**Combination of 4 Problems**				
Affordability & Proximity & Permission & Support/Companionship	23.19	(23.09—23.29)	27.15	(27.05—27.26)
**Combination of 3 Problems**	**15.55**	**(15.47—15.63)**	**16.80**	**(16.71—16.89)**
Affordability & Proximity & Permission	3.84	(3.79—3.88)	4.80	(4.75—4.85)
Affordability & Proximity & Support/Companionship	8.97	(8.9—9.03)	9.01	(8.95—9.08)
Affordability & Permission & Support/Companionship	1.09	(1.07—1.12)	1.20	(1.18—1.23)
Proximity & Permission & Support/Companionship	1.65	(1.62—1.68)	1.79	(1.76—1.82)
**Combination of 2 Problems**	**17.10**	**(17.01—17.19)**	**17.58**	**(17.49—17.67)**
Affordability & Proximity	5.86	(5.8—5.91)	5.94	(5.88—5.99)
Affordability & Permission	1.77	(1.74—1.8)	1.90	(1.87—1.93)
Affordability & Support/Companionship	1.42	(1.39—1.44)	1.25	(1.23—1.28)
Proximity & Permission	0.88	(0.85—0.9)	1.23	(1.21—1.26)
Permission & Support/Companionship	0.62	(0.6—0.63)	0.57	(0.55—0.59)
Proximity & Support/Companionship	6.57	(6.51—6.63)	6.68	(6.62—6.74)
**1 problem**	**16.63**	**(16.55—16.72)**	**15.28**	**(15.19—15.36)**
Affordability only	4.45	(4.40—4.50)	3.49	(3.45—3.53)
Proximity only	5.83	(5.78—5.89)	6.60	(6.54—6.66)
Permission only	1.69	(1.66—1.72)	1.47	(1.44—1.49)
Support/Companionship only	4.66	(4.61—4.71)	3.72	(3.68—3.77)
**No problem**	**27.53**	**(27.42—27.63)**	**23.19**	**(23.09—23.29)**

Among the combination of two problems, the problem of proximity and support/companionship had the highest share and it reduced from 6.68% (95% CI: 6.62—6.74) in 2016 to 6.57% (95% CI: 6.51—6.63) in 2021 followed by affordability and proximity: 5.94% (95% CI: 5.88—5.99) in 2016 to 5.86% (95% CI: 5.8—5.91) in 2021. In case of a single problem category, the share slightly increased from 15.28% (95% CI: 15.19—15.36) in 2016 to 16.63% (95% CI: 6.55—16.72) in 2021. The problem of proximity emerged as a major problem, followed by support/companionship and affordability.

A higher percentage of women in rural areas reported that they were facing all four PAHC, and it was reduced to 26.55% (95% CI: 26.44–26.67) in 2016 from 30.44% (30.31—30.56) in 2021. A higher percentage of women in rural areas than in urban areas reported that proximity is the major problem. This reduced from 7.21% (95% CI: 7.14—7.28) in 2016 to 6.12% (95% CI: 6.05—6.18) in 2021. But, affordability increased from 3.18% (95% CI: 3.13—3.23) in 2016 to 3.96% (95% CI: 3.91—4.01) in 2021 (Supplementary Table 4).

### Variance partitioning coefficient

The variance partitioning coefficient does not vary much for reporting of any problem for all of four indicators of the study. In 2016, states contributed 43% of the variation in affordability, and 46% was contributed by clusters. In 2021, for affordability, we observe that state variation contributes to 53% of the total variation, followed by clusters at 40% and districts at 7%. For proximity, the contribution of states to the total variation has changed from 32% in 2016 to 41% in 2021; and that of 57% from clusters in 2016 to 51% in 2021. For permission, the contribution of states in terms of variation changed from 29% in 2016 to 26% in 2021, and 57% from clusters in 2016 to 62% in 2021. For support/companionship, the contribution of states to the total variation changed from 29% in 2016 to 44% in 2021; and that of 59% from clusters in 2016 to 49% in 2021 (Supplementary Fig. 1).

### District variation in problems of accessing healthcare

**Fig. 1:**
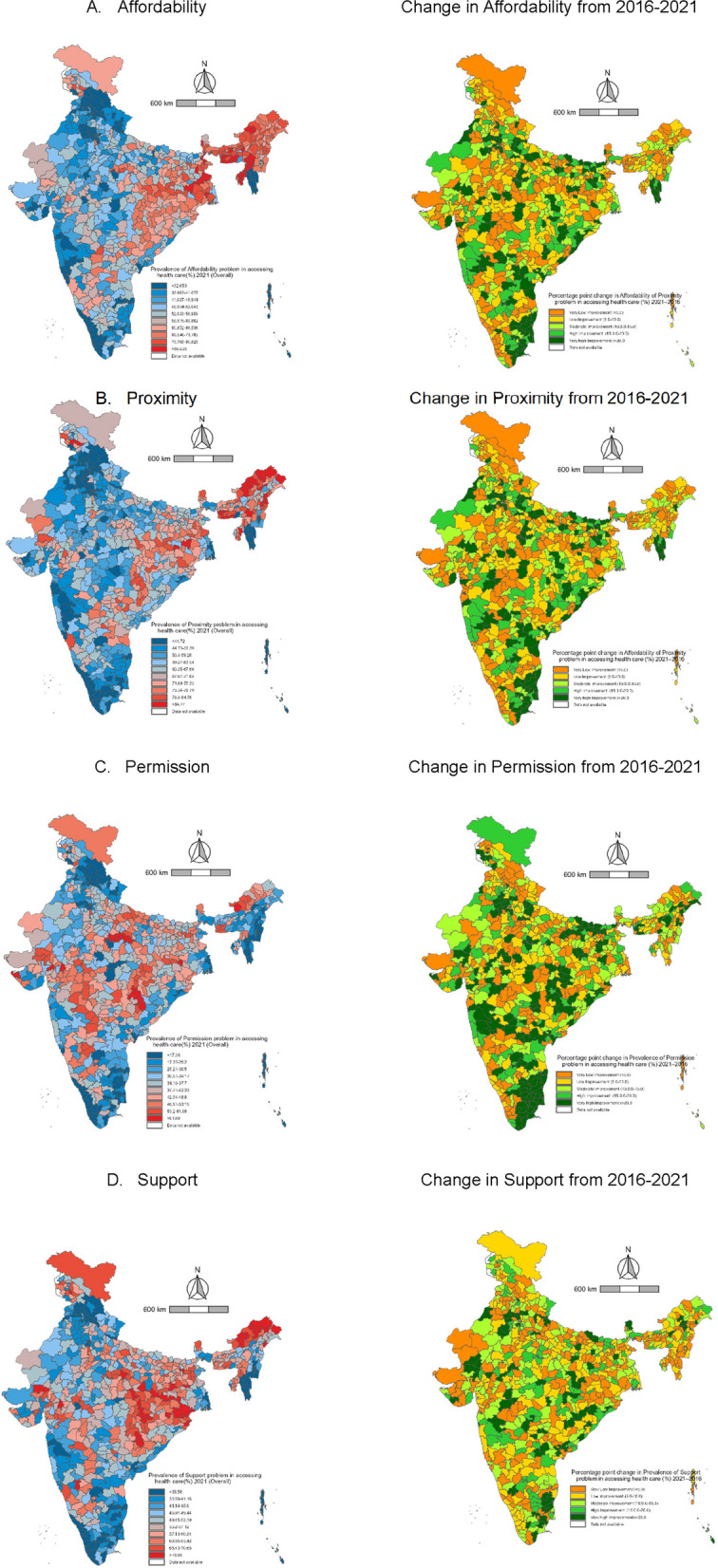
Maps of India illustrating the district-level percentage of women aged 15–49 years who reported problems in accessing healthcare in 2021 and the absolute change in prevalence from 2016 to 2021

As of 2021, affordability remains a significant issue in eastern India, particularly in the northeastern states of Arunachal Pradesh, and Assam, and the western regions of West Bengal, where over 47% of individuals reported it as a major concern. In parts of southern Chhattisgarh, southern Jharkhand, and northern Odisha, between 27 and 47% of the population similarly identified affordability as a key issue (Fig. 1). Proximity was also a substantial concern, with more than 50% of individuals in these regions noting it as a problem, especially concentrated in Arunachal Pradesh. In certain parts of Telangana, western West Bengal, and southern Jharkhand, 34% to 50% of women highlighted proximity as a major challenge. Similarly, 34% to 43% of women in northern Madhya Pradesh and parts of Himachal Pradesh reported proximity as a significant barrier. Issues of permission affected 24% to 26% of women in parts of Himachal Pradesh, Uttar Pradesh, Bihar, western West Bengal, central Maharashtra, and Karnataka. Notably, more than 25% of women in Arunachal Pradesh faced permission-related challenges. Finally, support/companionship was a major issue for over 34% of women in Arunachal Pradesh, western West Bengal, eastern Bihar, Jharkhand, and parts of Chhattisgarh. In central India, particularly in Madhya Pradesh, Rajasthan, parts of Maharashtra and Gujarat, and southern Karnataka, 20% to 23% of women also reported challenges related to support/companionship.

In 2016 (Supplementary Fig. 2), over 47% of women reported affordability as a problem in more districts of Odisha, primarily in southern Odisha, parts of Tamil Nadu, Andhra Pradesh, northern Bihar, Uttar Pradesh, Meghalaya, and Mizoram. Proximity was a problem for over 50% of women in all districts of Arunachal Pradesh, as well as in southern Jharkhand, northern Bihar, and northern Uttar Pradesh. Permission issues affected more than 33% of women in northern Bihar, northern Uttar Pradesh, and most districts of Tamil Nadu, Andhra Pradesh, Telangana, and parts of Maharashtra and Madhya Pradesh. Support/companionship was a major concern in all districts of Arunachal Pradesh, northern Bihar, northern Uttar Pradesh, Odisha, southern Jharkhand, and parts of Himachal Pradesh.

Geographic clustering of women identifying affordability, proximity, permission, and support/companionship as problems followed a similar pattern in both 2016 and 2021. However, in 2021, 60% to 66% of women additionally reported affordability as an issue in southern Karnataka and northern Kerala, with more districts in southern Odisha affected. Similarly, 71% to 76% of women reported proximity as a problem in northern Tamil Nadu and eastern Karnataka. Permission issues were identified by 42% to 53% of women in central Maharashtra, Karnataka, parts of Gujarat, and southern Chhattisgarh. Similar patterns were observed for support/companionship-related challenges.

Rural–urban differentials also followed a similar trend to the overall pattern. Affordability was a major problem than other three problems in the urban areas in the majority of the districts of Chhattisgarh, Odisha, West Bengal and northeastern states except Mizoram. Proximity has improved in all the districts in India and still, it is a major problem in the majority of the districts of Arunachal Pradesh and a few districts of West Bengal and Bihar. Permission was improved in all the districts and still, more than 35% of the women in central India reported that getting permission to go to health care is a major problem (Supplementary Figs. 3 & 4).

### District-level change in problems of accessing healthcare between 2016 and 2021

The district-level change among all the four indicators was positive (Fig. 1). The median district prevalence of affordability problem has reduced from 56.92% in 2016 to 54.04% in 2021. For the proximity problem, the median reduced from 67.88% to 62.66% in 2021. The median prevalence of permission reduced from 37.77% in 2016 to 34.70% in 2021. For support/companionship problems, the median reduced to 51.70% in 2021 from 53.25% in 2016. 333 districts among 720 districts have experienced more than a 10% absolute decline from 2016 in affordability problem. Similarly, for accessibility, permission and support/companionship, 304, 385 and 330 districts have experienced more than a 10% reduction respectively.

The largest change in the affordability problem was observed in the Northern districts of Tamil Nadu and Odisha, a few districts of Bihar and Madhya Pradesh. Similarly, for proximity, the highest changes were observed in north Gujarat, most of the districts in Uttar Pradesh and Bihar. The largest change in permission was observed in most of the districts of Tamil Nadu and Andhra Pradesh.

Rural–urban differentials also followed a similar trend to the overall pattern. Among all the indicators the largest change was observed in permission problem.

## Discussion

Our study has four major findings. First, proximity was identified as the major problem in accessing healthcare among women 15–49 years of age in India. This was more prevalent in Northeastern states, majority districts of West Bengal, Odisha, Telangana, Chattisgarh and Bihar. The combination of proximity and affordability had a higher prevalence than other combinations, although it declined from 2016 to 2021. Rural and urban differentials also followed the same pattern. Second, at all India level and also in rural–urban differentials, major changes were observed in all four indicators of PAHC. Third, all four problems were highly prevalent in northeastern and eastern states, with problems of permission and support/companionship being highly prevalent than other two in northern and western states, and support/companionship being identified as a major problem in southern states. Positive changes in all four problems were higher in eastern and southern states. Fourth, except permission, the state contribution in the variance partitioning coefficient has increased and the contribution of districts and clusters has decreased.

Our findings should be interpreted in the light of a few limitations. First, 54 clusters were excluded from 2016 due to the reallocation of clusters to match with 2021 districts. Second, the data for Andhra Pradesh was collected from 13 districts and to match with 2021 we converted to 26 districts and the results from these districts are underpowered. However, the standard error of the estimates was small which indicates these estimates are reliable. Third, the assessment of PAHC in NFHS is a subjective matter. However, as we are reporting user perspective, the subjectivity of such reporting may not affect the policy relevance of the same. Fourth, this study primarily focuses on healthcare access but may not address healthcare quality, which is equally critical for better health outcomes. Fifth, while we recommend decentralization of policy making, the paper may not deeply analyse the political or logistic challenges in implementing such an approach in lagging states. 

Studies have shown that proximity is one of the major problems in accessing healthcare in India, especially in rural areas [34]. Particularly in the Northeastern states, the health infrastructure is poor in comparison with other states. For example, the number of Sub-centres, PHCs and CHCs has declined in all the states except Odisha and Telangana from 2005 to 2022 [35, 36]. Additionally, the annual report of the Ministry of Health and Family Welfare also stated that the Northeastern states, Bihar and Odisha, lack sufficient healthcare facilities, making proximity a more substantial barrier than affordability [37]. The persistent problem of proximity, despite the implementation of national health policies, underscores the role of geographic remoteness and lower density of health care providers. In contrast, states like Tamil Nadu and Kerala, which have a strong healthcare system and higher female education, have reported less number of problems, indicating the benefits of long-term investments in social development. A slight reduction in the proximity and affordability problems observed in our study can be attributed to health care policies like the Ayushman Bharat initiative [38–40]. However, improvements are slower to materialise due to persistent implementation challenges.

At all-India level, we observed that for all four barriers to access, the percentage of individuals reporting them as a major problem have reduced between 2016 and 2021. The introduction of healthcare expansion initiatives such as Ayushman Bharat that encompasses financial protetction, digitisation, as well as primary care, may have contributed to the reduction of people reporting PAHC . However, moderate issues of access may not mean lack of access altogether; but could play a great role in delayed healthcare seeking. Such delays will imply a lack of efficiency in policies to provide healthcare access in a timely manner rather than their availability altogether. Efficiency in healthcare policies extends beyond ensuring the mere availability of services; it includes the timely and effective delivery of care to those in need [41]. While initiatives like Ayushman Bharat have significantly improved access by addressing key problems, delays in healthcare-seeking behaviour due to moderate access issues underscore persistent gaps in the operational efficiency of the healthcare system [42]. These inefficiencies can result in the underutilization of available services or exacerbate health outcomes despite improvement in access. To address these challenges, it is important to enhance responsiveness and outreach, thereby bridging the systematic gap and improving the overall impact of policy.

In the northern region, a greater percentage of women reported permission and support/companionship as major problems than affordability and proximity. Such observations can be explained by strong patriarchal norms as well as safety issues faced by females in these regions. Previous studies also revealed that the percentage of women who are part of household decision-making and those who make decisions on their own healthcare is low among the states where the prevalence of permission and support/companionship problems is high [14, 43–46]. NCRB estimates show that crimes against women are high in these regions too [20]. Further, differences in the importance of health seeking by gender may also contribute to such barriers for females. A study conducted by Dupas and Jian shows that households are willing to spend more to seek better care for males than females and also travel more distances to get better care for males than females [47]. This suggests that men make key decisions regarding healthcare access which limits women’s ability to seek care independently [43]. The persistence of such strong cultural barriers suggests that improvement in affordability and infrastructure alone may not eliminate the access gap unless included with gender-sensitive interventions that improve autonomy.

We also observed that the relative contribution of districts in explaining the geographic variation in reported problems of access to healthcare, but between 2016 and 2021, the contribution of states has increased while that of clusters has reduced. Usually, when the role of states in variation is increasing, it implies an underlying difference in the effectiveness of policy initiatives, as health is a state subject [48]. These variations can be partly linked to differences in implementation of policies and socio-economic development. For example inclusion of financial measures like ownership of assets and ownership of bank accounts can help understand and access the health care.

Given that the context of accessibility issues requires tailored policy responses, states need to look closer into the reasons for low accessibility instead of adopting national guidelines for health system improvements [49]. This underscores the decentralization approach to healthcare policy-making where policies are formulated to address state-specific challenges and priorities. The regions where proximity is a barrier like the northeastern and eastern regions, the infrastructure should be improved. In the regions where permission is a problem, gender sensitive policies should be implemented to overcome. The coverage of the financial protection programmes like Ayushman Bharat should be increased in the areas where affordability is a major problem.

## Conclusion

The study analyses the district-level variations and changes in four major problems in access to health care reported by women in India aged 15–49 years between 2016 to 2021. Proximity is a major problem in northeastern and eastern regions, while affordability remains as a persistent and key problem in several regions. Cultural problems like permission and support/companionship are major problems in Western and northern states. Although the study observed positive changes in all four problems across the districts, persistent geographic and socio-cultural challenges call for decentralised and gender-sensitive policies. Improving health care infrastructure in the underserved districts, combined with health care financing, is critical in improving access and ultimately improving health outcomes among women in India.

## Supplementary Information

Supplementary Material 1.

## Data Availability

The datasets are publically available at [https://dhsprogram.com].

## Abbreviations

AC: Assembly Constituency
AP: Andhra Pradesh
CEO: Chief Electoral Officer
DHS: Demographic and Health Surveys Program
ESS: Effective Sample Size
MCMC: Markov Chain Monte Carlo
NFHS: National Family Health Survey
PAHC: Problems in Accessing Health Care
UTs: Union Territories
