# Supplementary material for: Problems in accessing healthcare among women in India: a district-level change analysis, 2016–2021
Source: BMC Public Health. 2026 Feb 6;26:544. doi: 10.1186/s12889-026-26392-7 (PMC12888451; doi:10.1186/s12889-026-26392-7)
Supplement: Supplementary file 1 — Supplementary Material 1. [file 12889_2026_26392_MOESM1_ESM.docx]

**Supplementary Table 1:** Markov Chain Monte Carlo (MCMC) model output for Problems in Accessing Healthcare (PAHC) in 2016 and 2021

| **Survey/outcome** | **Geographic level** | **Coefficient** | **Std Dev** | **Effective Sample Size** | **Credible interval** | **Other MCMC** | |
| --- | --- | --- | --- | --- | --- | --- | --- |
| **NFHS-5 Affordability** | **Constant** | -0.19 | 0.05 | 3 | (-0.29 - -0.11) | B DIC | 763262.17 |
|  | **State** | 1.79 | 0.46 | 2988 | (1.09 - 2.83) | dbar | 738400.59 |
|  | **District** | 0.23 | 0.01 | 488 | (0.21 - 0.26) | dbar | 24861.58 |
|  | **Cluster** | 1.37 | 0.02 | 786 | (1.34 - 1.4) | thetabar | 788123.76 |
| **NFHS-4 Affordability** | **Constant** | 0.34 | 0.06 | 4 | (0.2 - 0.42) | effective no. of pars | 727654.85 |
|  | **State** | 1.31 | 0.35 | 955 | (0.78 - 2.13) | B DIC | 704091.77 |
|  | **District** | 0.37 | 0.02 | 374 | (0.32 - 0.41) | dbar | 23563.09 |
|  | **Cluster** | 1.40 | 0.02 | 879 | (1.37 - 1.43) | thetabar | 751217.94 |
| **NFHS-5 Proximity** | **Constant** | 0.28 | 0.07 | 3 | (0.17 - 0.39) | effective no. of pars | 735237.66 |
|  | **State** | 1.34 | 0.37 | 687 | (0.8 - 2.23) | B DIC | 710005.26 |
|  | **District** | 0.26 | 0.02 | 126 | (0.23 - 0.3) | dbar | 25232.40 |
|  | **Cluster** | 1.65 | 0.02 | 701 | (1.61 - 1.69) | thetabar | 760470.06 |
| **NFHS-4 Proximity** | **Constant** | 0.42 | 0.02 | 13 | (0.37 - 0.46) | effective no. of pars | 665662.33 |
|  | **State** | 1.08 | 0.29 | 2365 | (0.65 - 1.75) | B DIC | 641918.24 |
|  | **District** | 0.37 | 0.02 | 142 | (0.33 - 0.42) | dbar | 23744.09 |
|  | **Cluster** | 1.95 | 0.02 | 896 | (1.9 - 1.99) | thetabar | 689406.42 |
| **NFHS-5 Permission** | **Constant** | -1.16 | 0.03 | 5 | (-1.21 - -1.09) | effective no. of pars | 730447.11 |
|  | **State** | 0.66 | 0.19 | 349 | (0.39 - 1.13) | B DIC | 705712.99 |
|  | **District** | 0.30 | 0.02 | 441 | (0.27 - 0.34) | dbar | 24734.12 |
|  | **Cluster** | 1.54 | 0.02 | 960 | (1.5 - 1.57) | dbar | 755181.23 |
| **NFHS-4 Permission** | **Constant** | -0.78 | 0.06 | 4 | (0.9 - -0.68) | thetabar | 691903.95 |
|  | **State** | 0.87 | 0.24 | 297 | (0.51 - 1.46) | effective no. of pars | 668158.78 |
|  | **District** | 0.44 | 0.03 | 524 | (0.39 - 0.5) | B DIC | 23745.18 |
|  | **Cluster** | 1.72 | 0.02 | 672 | (1.68 - 1.76) | dbar | 715649.13 |
| **NFHS-5 Support** | **Constant** | -0.11 | 0.03 | 7 | (0.18 - -0.06) | thetabar | 796904.64 |
|  | **State** | 1.18 | 0.31 | 2379 | (0.71 - 1.9) | effective no. of pars | 771782.33 |
|  | **District** | 0.18 | 0.01 | 235 | (0.16 - 0.21) | B DIC | 25122.31 |
|  | **Cluster** | 1.30 | 0.01 | 939 | (1.27 - 1.33) | dbar | 822026.95 |
| **NFHS-4 Support** | **Constant** | -0.15 | 0.04 | 3 | (0.22 - -0.08) | thetabar | 760129.27 |
|  | **State** | 0.66 | 0.18 | 361 | (0.39 - 1.09) | effective no. of pars | 736189.81 |
|  | **District** | 0.27 | 0.02 | 277 | (0.24 - 0.3) | B DIC | 23939.45 |
|  | **Cluster** | 1.36 | 0.02 | 938 | (1.33 - 1.39) | dbar | 784068.72 |

**Supplementary figure 1:** Geographic variance partitioned between states, districts, and clusters for Problems in Accessing Healthcare (PAHC) in 2016 and 2021.

**Supplementary Table 2:** Sample size (N) and Weighted percentage of women aged 15-49 years Problems in Accessing Healthcare (PAHC) in India, Urban, 2016-2021

| **Variables** | **2021** | | **2016** | |
| --- | --- | --- | --- | --- |
|  | N | % | N | % |
| **Affordability** |  |  |  |  |
| *No problem* | 1,06,333 | 60.43 | 1,14,715 | 55.32 |
| *Any problem* | 73,202 | 39.57 | 89,889 | 44.68 |
| **Proximity** |  |  |  |  |
| *No problem* | 1,02,675 | 58.28 | 1,06,864 | 51.79 |
| *Any problem* | 76,860 | 41.72 | 97,740 | 48.20 |
| **Permission** |  |  |  |  |
| *No problem* | 1,28,868 | 72.34 | 1,38,983 | 65.2 |
| *Any problem* | 50,667 | 27.65 | 65,621 | 34.80 |
| **Support/Companionship** |  |  |  |  |
| *No problem* | 1,10,799 | 63.08 | 1,21,096 | 58.87 |
| *Any problem* | 68,736 | 36.92 | 83,508 | 41.12 |

**Supplementary Table 3:** Sample size (N) and Weighted percentage of women aged 15-49 years Problems in Accessing Healthcare (PAHC) in India, Rural, 2016-2021

| **Variables** | **2021** | | **2016** | |
| --- | --- | --- | --- | --- |
|  | N | % | N | % |
| **Affordability** |  |  |  |  |
| *No problem* | 2,33,830 | 44.13 | 1,98,969 | 39.92 |
| *Any problem* | 3,10,750 | 55.87 | 2,94,806 | 60.08 |
| **Proximity** |  |  |  |  |
| *No problem* | 1,84,225 | 35.97 | 1,37,375 | 28.85 |
| *Any problem* | 3,60,355 | 64.04 | 3,56,400 | 71.15 |
| **Permission** |  |  |  |  |
| *No problem* | 3,38,883 | 61.88 | 2,91,547 | 57.08 |
| *Any problem* | 2,05,697 | 38.12 | 2,02,228 | 42.93 |
| **Support/Companionship** |  |  |  |  |
| *No problem* | 2,45,360 | 46.43 | 2,09,429 | 43.18 |
| *Any problem* | 2,99,220 | 53.57 | 2,84,346 | 56.81 |

**Supplementary Table 4:** Percentage of women aged 15-49 years reported different types of Problems in Accessing Healthcare (PAHC) in all possible combinations in India, Urban vs Rural, 2016-2021

| **Combination of Problems** | Urban | | | | Rural | | | |
| --- | --- | --- | --- | --- | --- | --- | --- | --- |
|  | 2021 | 95% Confidence Interval | 2016 | 95% Confidence Interval | 2021 | 95% Confidence Interval | 2016 | 95% Confidence Interval |
| **Combination of 4 Problems** |  |  |  |  |  |  |  |  |
| Affordability & Proximity & Permission & Support/Companionship | 16.20 | (16.03 - 16.37) | 20.96 | (20.78 - 21.13) | 26.55 | (26.44 - 26.67) | 30.44 | (30.31 - 30.56) |
| **Combination of 3 Problems** |  |  |  |  |  |  |  |  |
| Affordability & Proximity & Permission | 3.20 | (3.12 - 3.28) | 4.14 | (4.05 - 4.22) | 4.15 | (4.09 - 4.2) | 5.15 | (5.09 - 5.21) |
| Affordability & Proximity & Support/Companionship | 5.97 | (5.86 - 6.08) | 5.77 | (5.67 - 5.87) | 10.41 | (10.33 - 10.49) | 10.73 | (10.65 - 10.82) |
| Affordability & Permission & Support/Companionship | 1.12 | (1.07 - 1.17) | 1.44 | (1.39 - 1.5) | 1.08 | (1.05 - 1.11) | 1.07 | (1.04 - 1.1) |
| Proximity & Permission & Support/Companionship | 1.32 | (1.27 - 1.38) | 1.58 | (1.52 - 1.63) | 1.81 | (1.78 - 1.85) | 1.90 | (1.86 - 1.94) |
| **Combination of 2 Problems** |  |  |  |  |  |  |  |  |
| Affordability & Proximity | 3.97 | (3.88 - 4.06) | 4.33 | (4.24 - 4.41) | 6.77 | (6.7 - 6.83) | 6.79 | (6.72 - 6.86) |
| Affordability & Permission | 2.19 | (2.12 - 2.26) | 2.56 | (2.5 - 2.63) | 1.56 | (1.53 - 1.6) | 1.55 | (1.51 - 1.58) |
| Affordability & Support/Companionship | 1.46 | (1.41 - 1.52) | 1.42 | (1.37 - 1.47) | 1.39 | (1.36 - 1.42) | 1.16 | (1.13 - 1.19) |
| Proximity & Permission | 0.77 | (0.73 - 0.81) | 1.18 | (1.13 - 1.23) | 0.93 | (0.9 - 0.95) | 1.26 | (1.23 - 1.29) |
| Permission & Support/Companionship | 0.67 | (0.63 - 0.71) | 0.72 | (0.69 - 0.76) | 0.59 | (0.57 - 0.61) | 0.49 | (0.47 - 0.51) |
| Proximity & Support/Companionship | 5.05 | (4.95 - 5.16) | 4.82 | (4.73 - 4.91) | 7.30 | (7.23 - 7.37) | 7.67 | (7.59 - 7.74) |
| **Only one problem** |  |  |  |  |  |  |  |  |
| Affordability only | 5.46 | (5.36 - 5.57) | 4.07 | (3.98 - 4.15) | 3.96 | (3.91 - 4.01) | 3.18 | (3.13 - 3.23) |
| Proximity only | 5.24 | (5.13 - 5.34) | 5.44 | (5.34 - 5.54) | 6.12 | (6.05 - 6.18) | 7.21 | (7.14 - 7.28) |
| Permission only | 2.19 | (2.12 - 2.26) | 2.22 | (2.16 - 2.28) | 1.46 | (1.42 - 1.49) | 1.07 | (1.04 - 1.1) |
| Support/Companionship only | 5.12 | (5.02 - 5.22) | 4.42 | (4.33 - 4.51) | 4.44 | (4.38 - 4.49) | 3.35 | (3.3 - 3.4) |

**Supplementary Figure 2:** Maps of India illustrating the district-level percentage of women aged 15-49 years reported Problems in Accessing Healthcare (PAHC) in 2016

| 1. 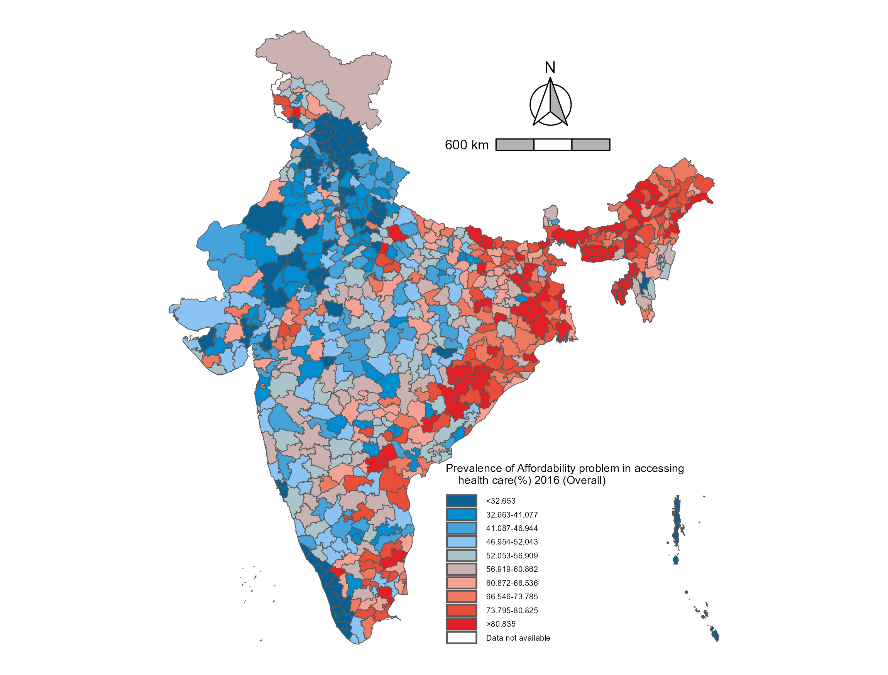Affordability | 1. 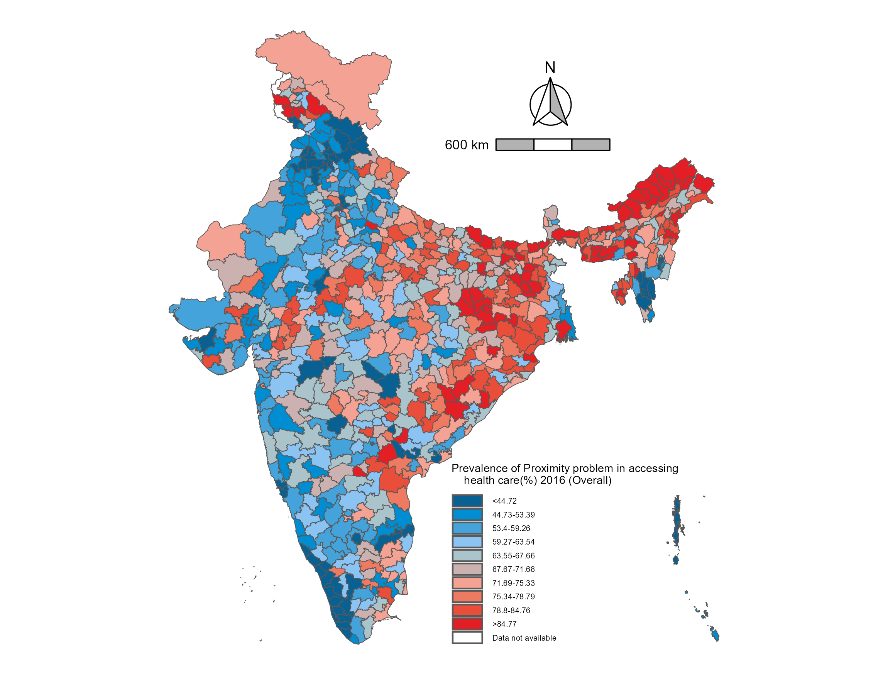Proximity |
| --- | --- |
|  |  |
| 1. 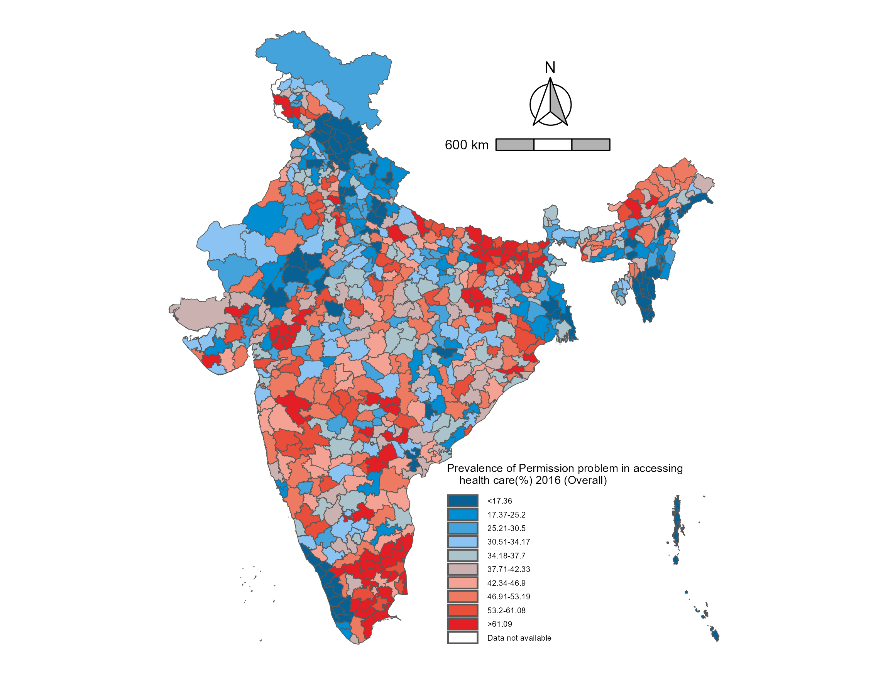Permission | 1. 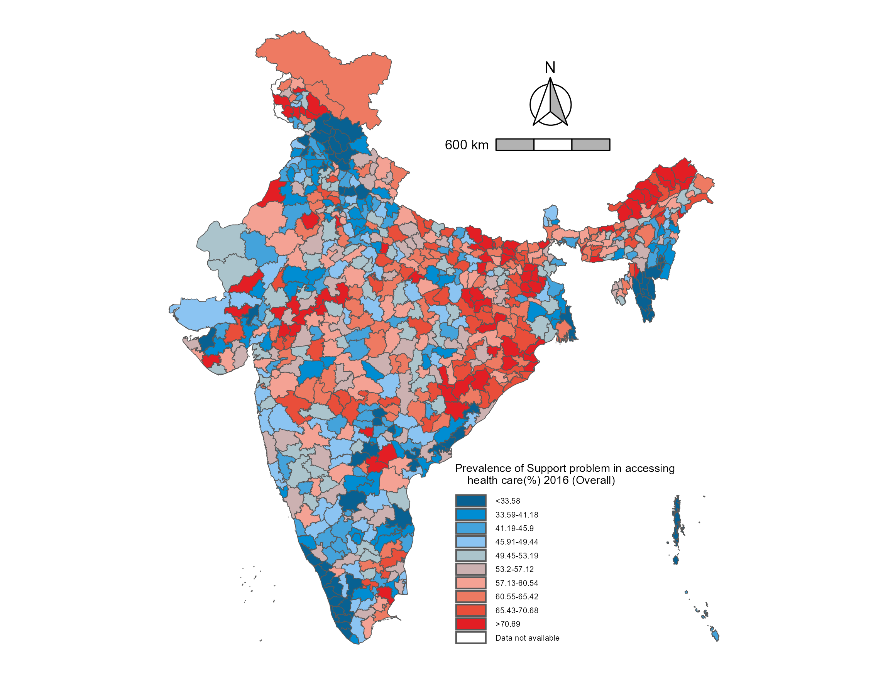Support |
|  |  |

**Supplementary Figure 3:** Maps of Urban India illustrating the district-level percentage of women aged 15-49 years reported Problems in Accessing Healthcare (PAHC) in 2021 and 2016 and the absolute change in prevalence from 2016 to 2021

| 1. Affordability, 2021 | Affordability, 2016 | Change in Affordability from 2016-2021 |
| --- | --- | --- |
| 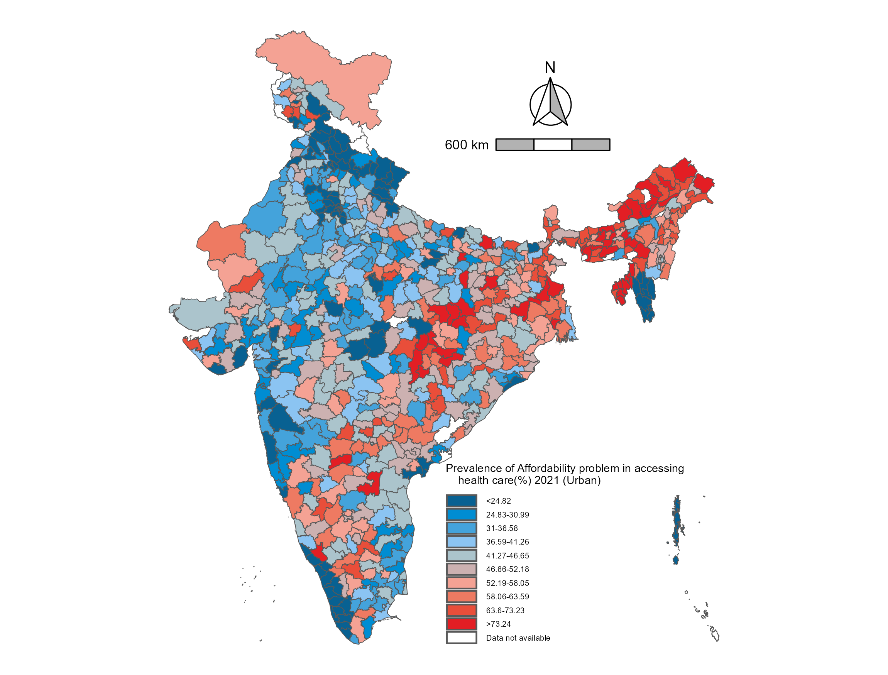 | 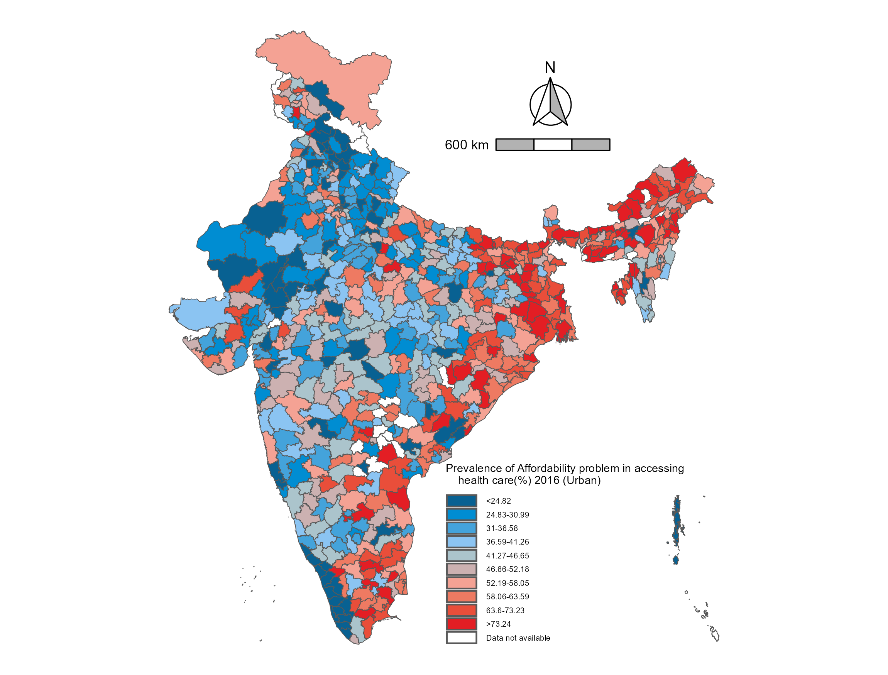 | 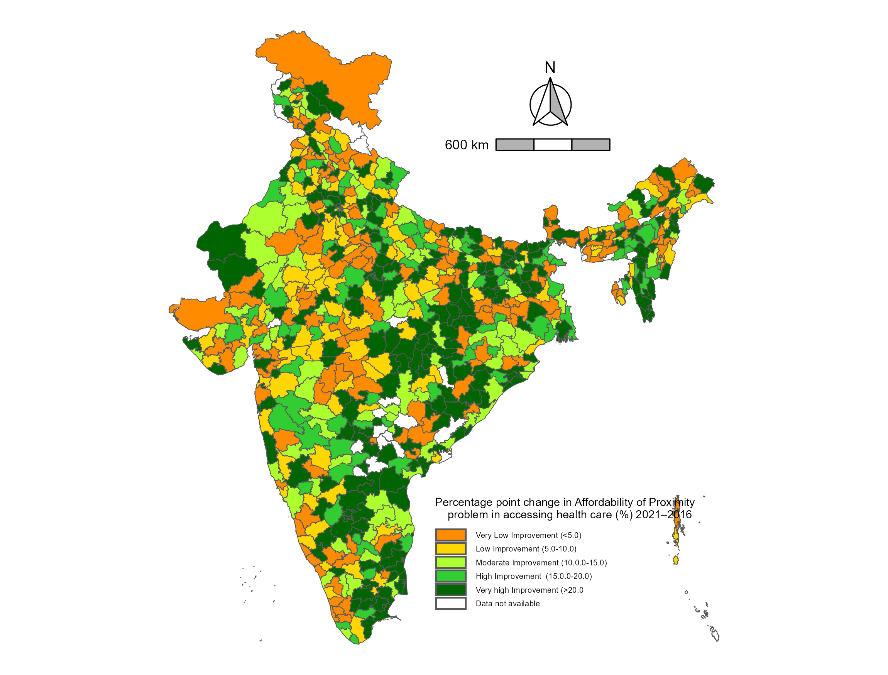 |
| 1. Proximity, 2021 | Proximity, 2016 | Change in Proximity from 2016-2021 |
| 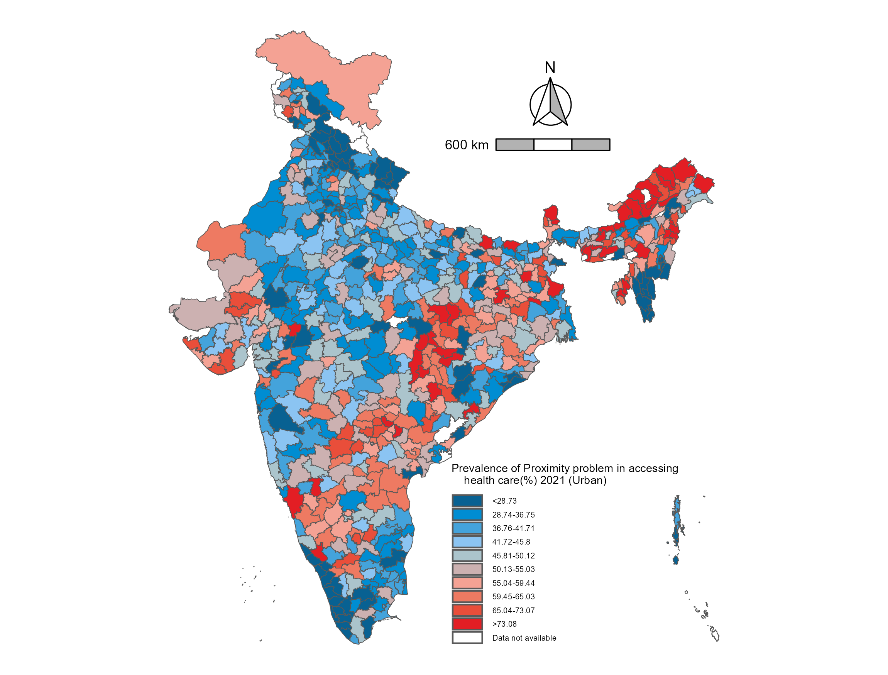 | 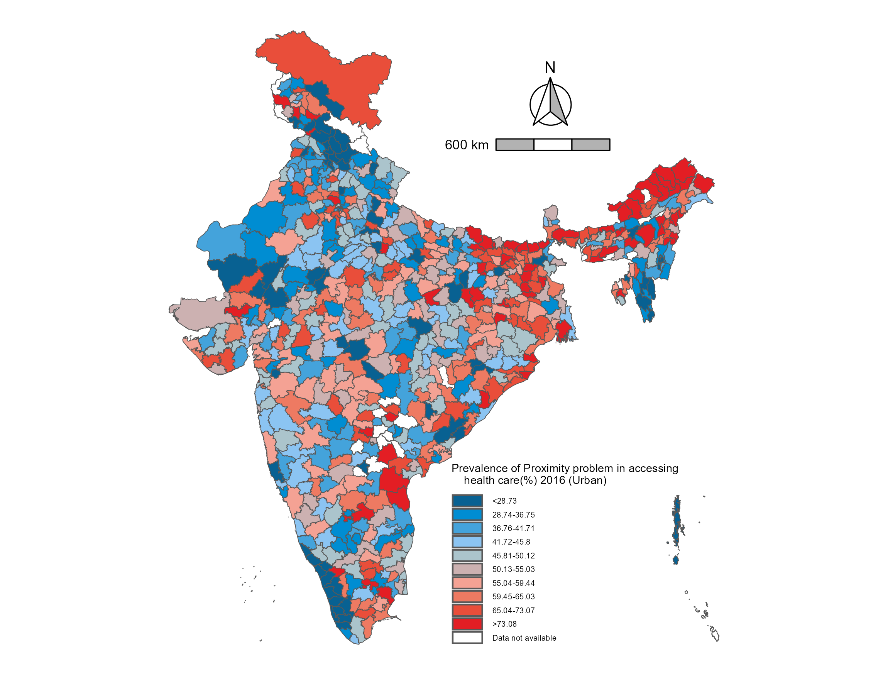 | 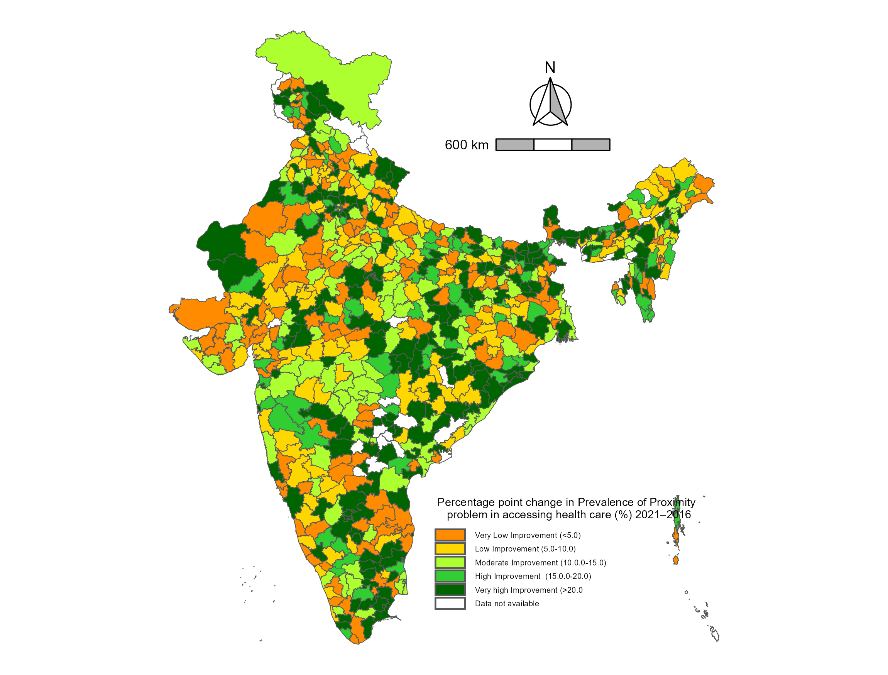 |

| 1. 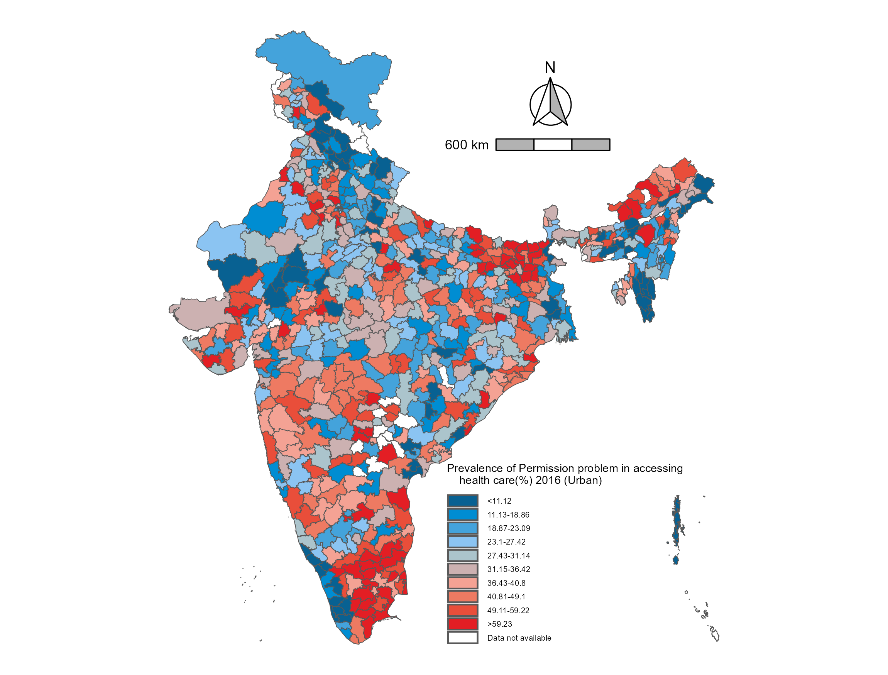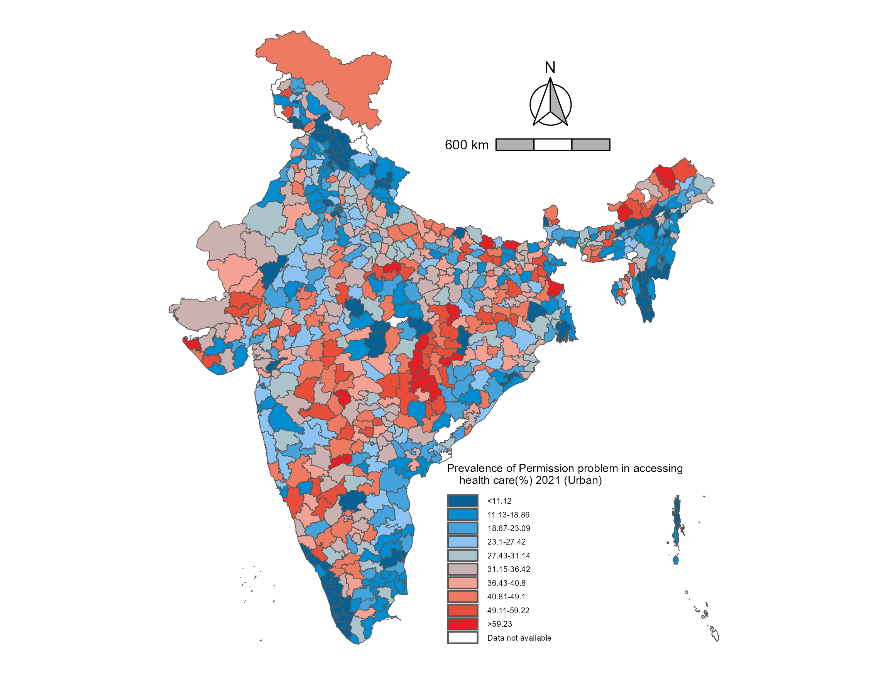Permission, 2021 | 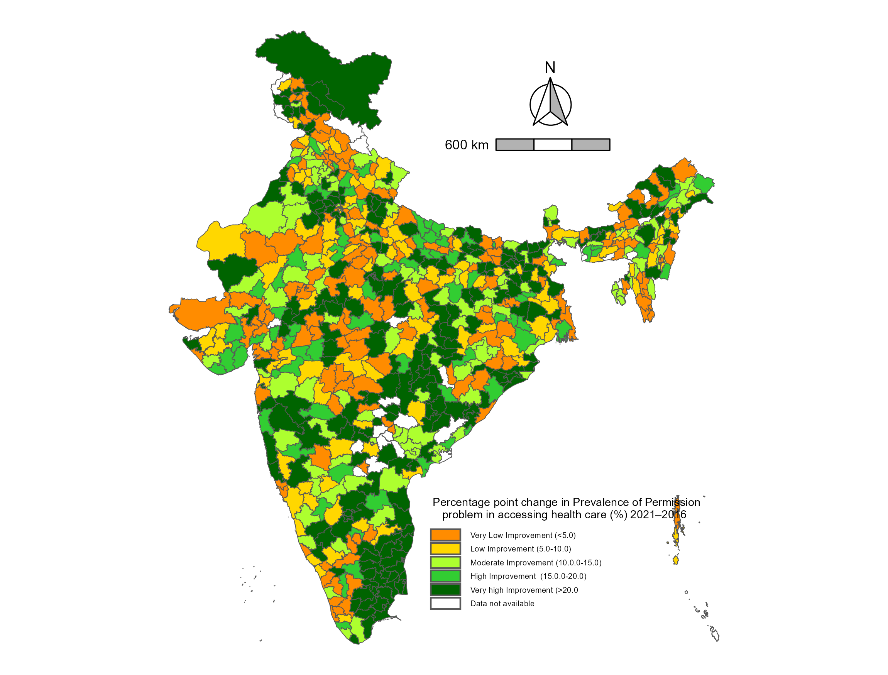Permission, 2016 | Change in Permission from 2016-2021 |
| --- | --- | --- |
|  |  |  |
| 1. Support, 2021 | Support, 2016 | Change in Support from 2016-2021 |
| 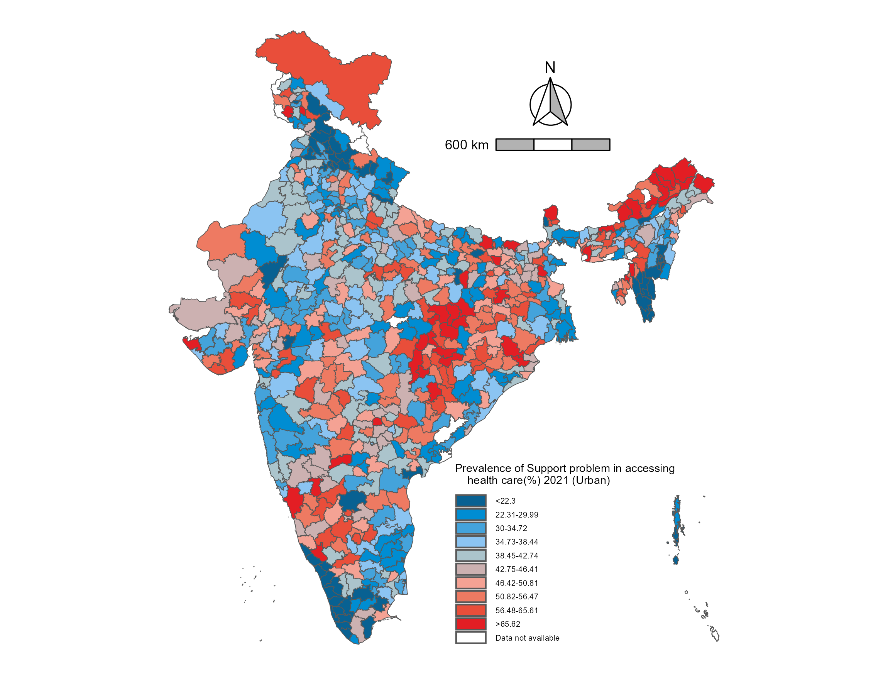 | 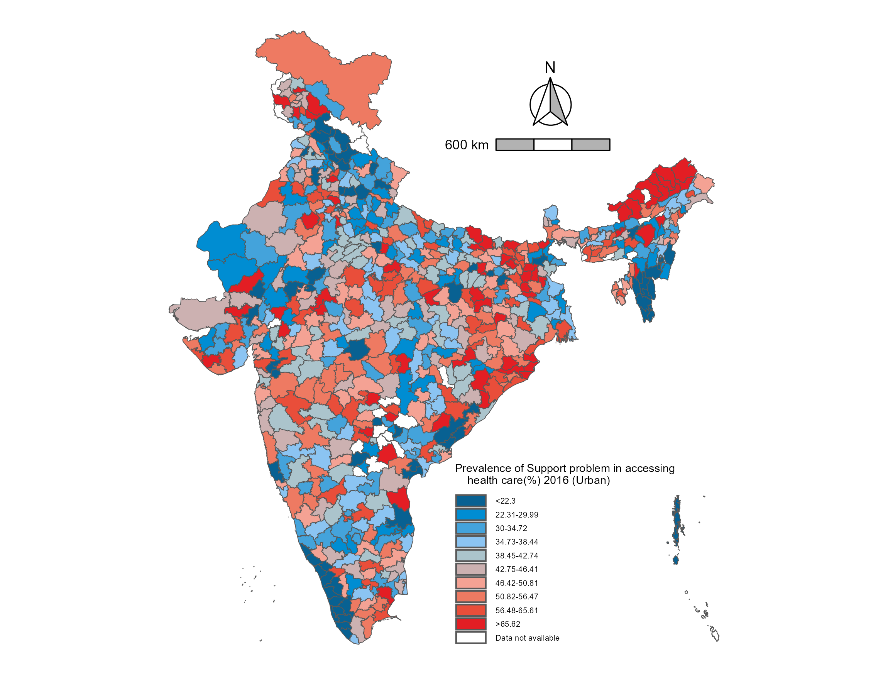 | 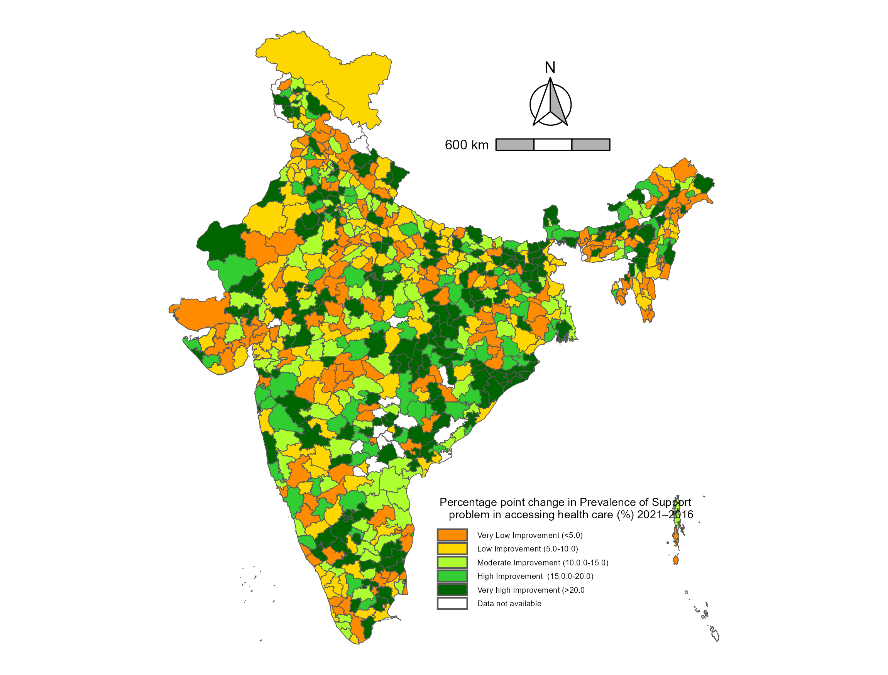 |

**Supplementary Figure 4:** Maps of Rural India illustrating the district-level percentage of women aged 15-49 years reported Problems in Accessing Healthcare (PAHC) in 2021 and 2016 and the absolute change in prevalence from 2016 to 2021

| 1. Affordability, 2021 | Affordability, 2016 | Change in Affordability from 2016-2021 |
| --- | --- | --- |
| 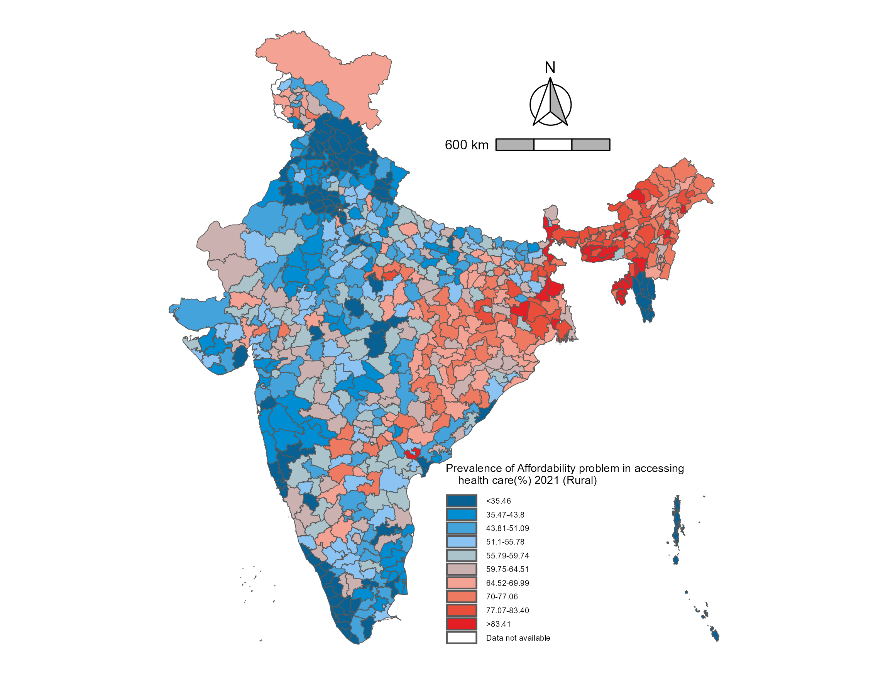 | 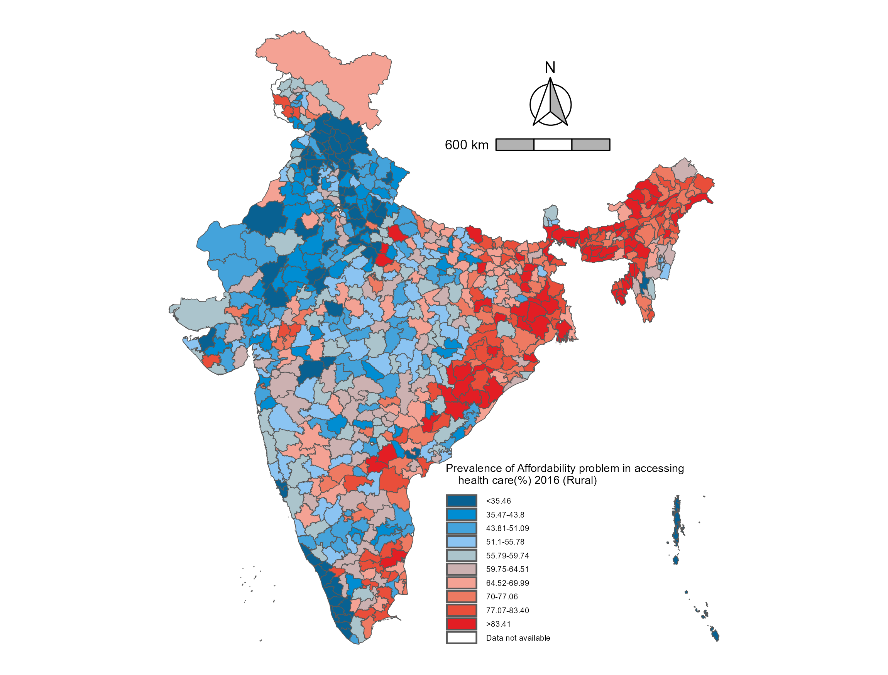 | 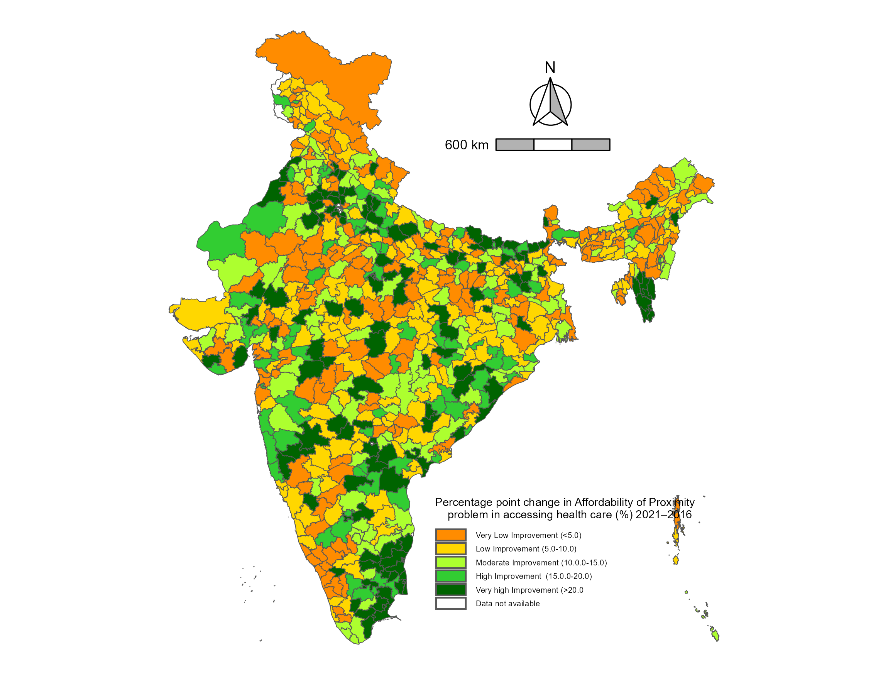 |
| 1. Proximity, 2021 | Proximity, 2016 | Change in Proximity from 2016-2021 |
| 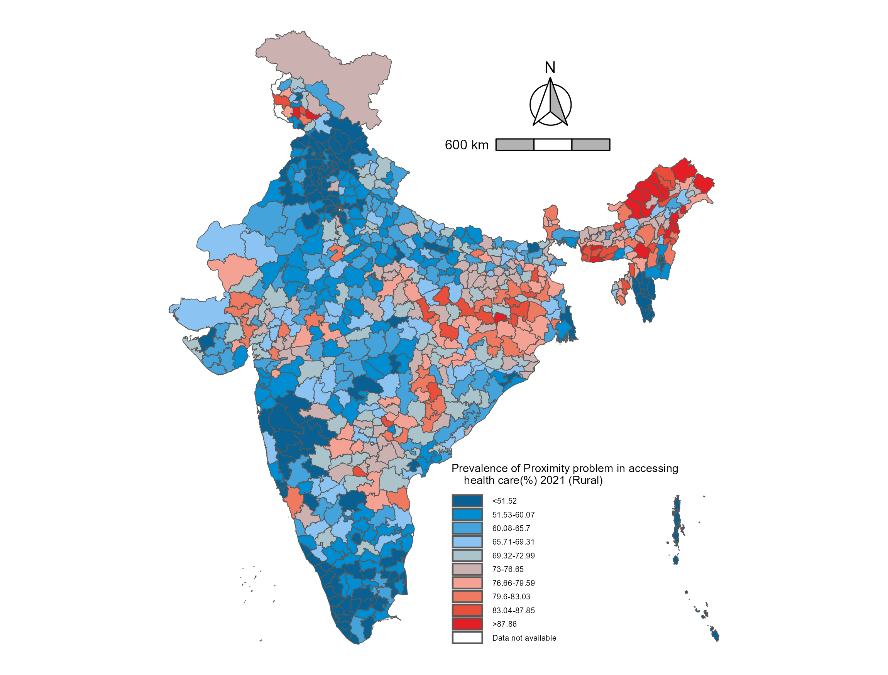 | 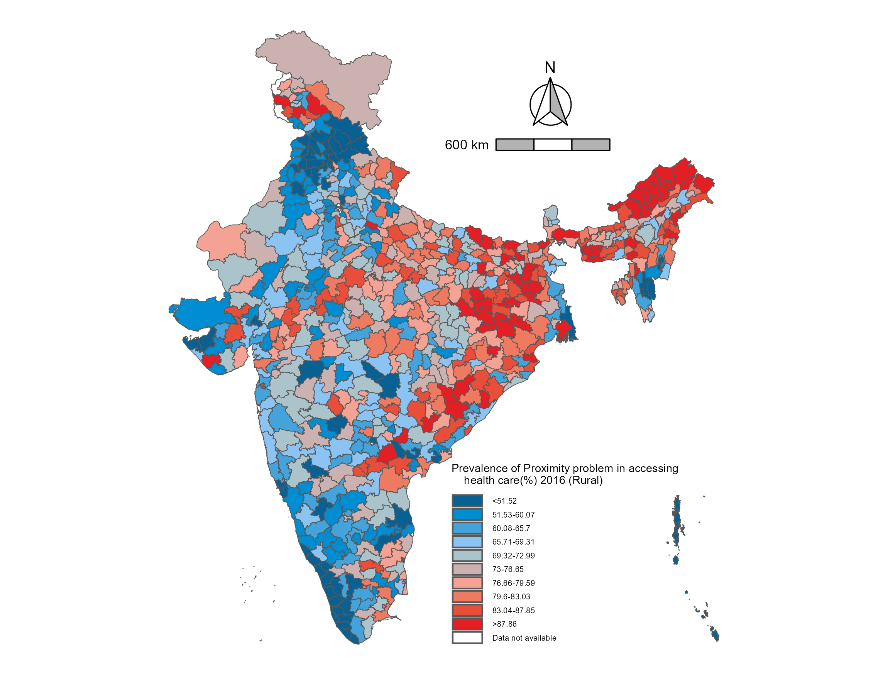 | 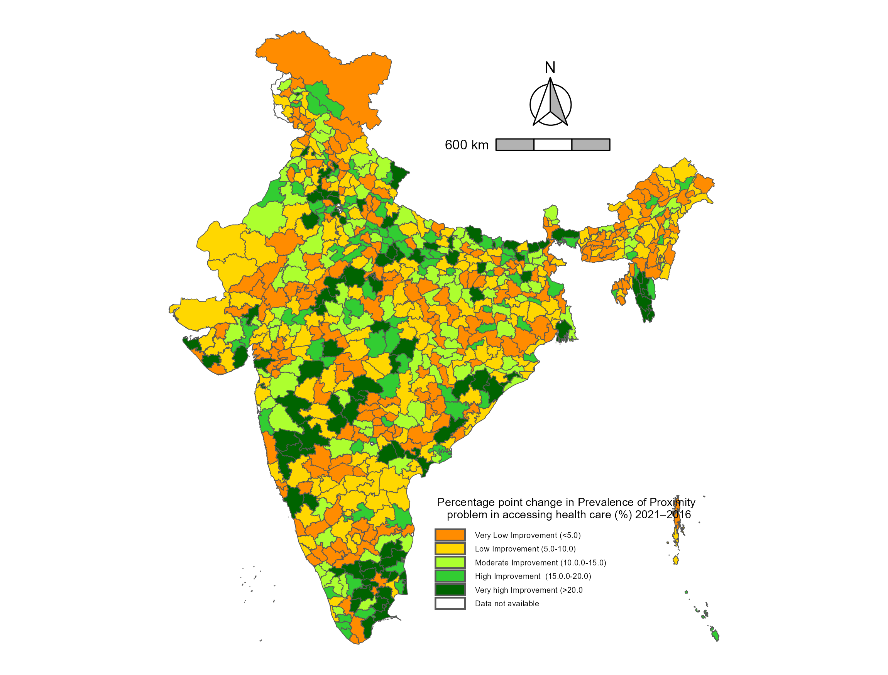 |

| 1. Permission, 2021 | Permission, 2016 | Change in Permission from 2016-2021 |
| --- | --- | --- |
| 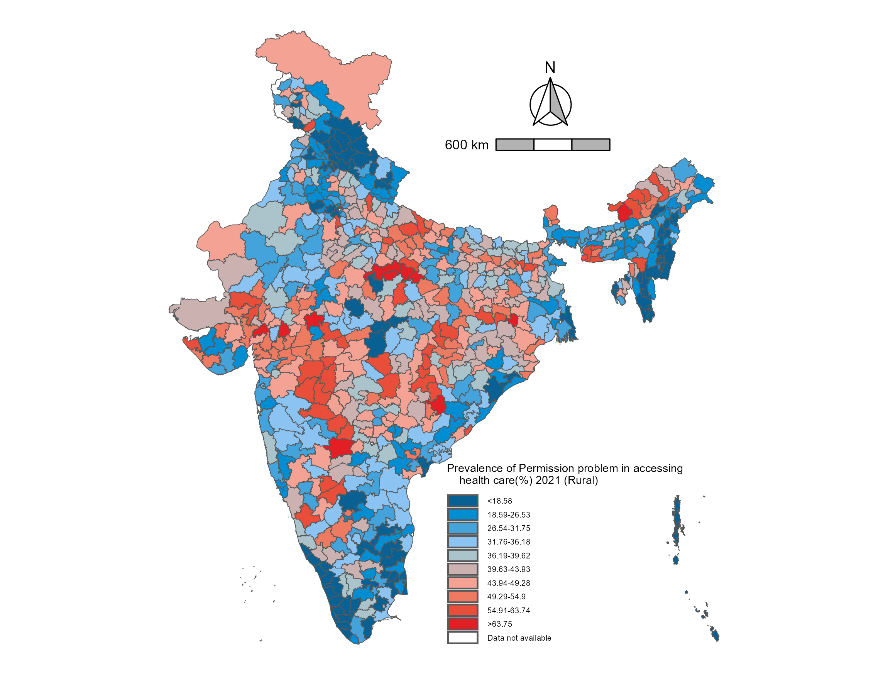 | 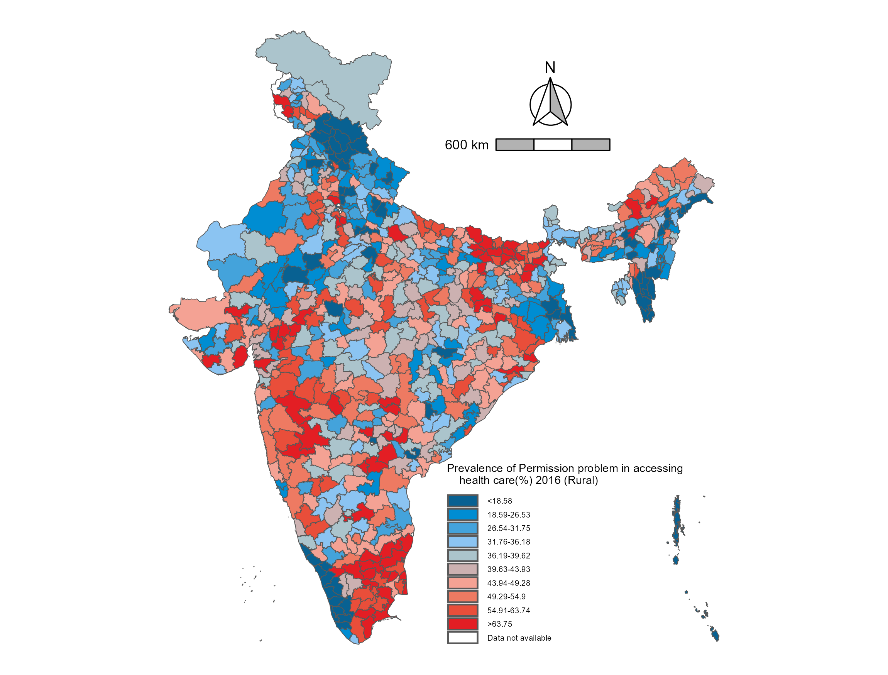 | 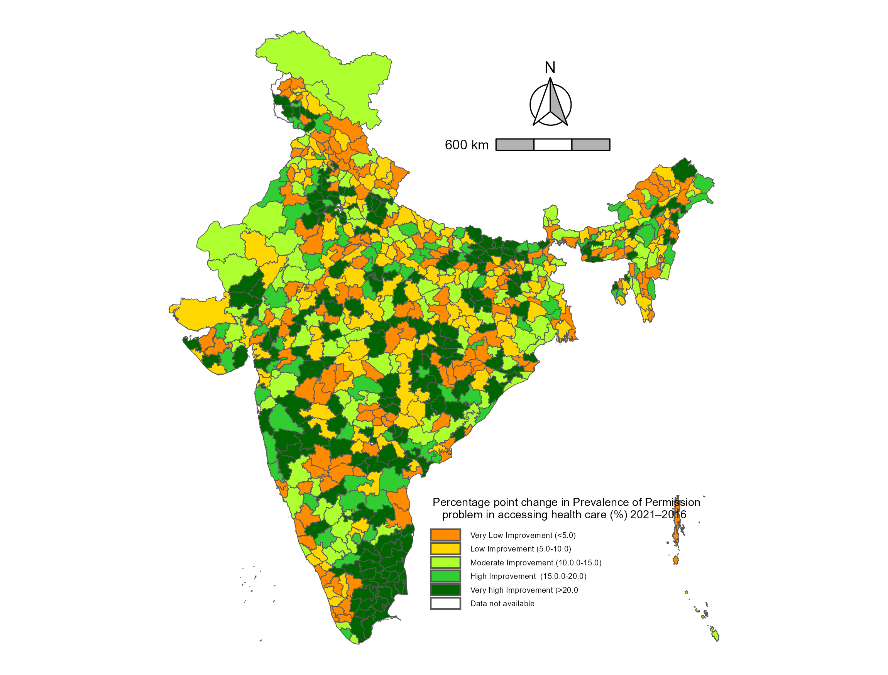 |
| 1. Support, 2021 | Support, 2016 | Change in Support from 2016-2021 |
| 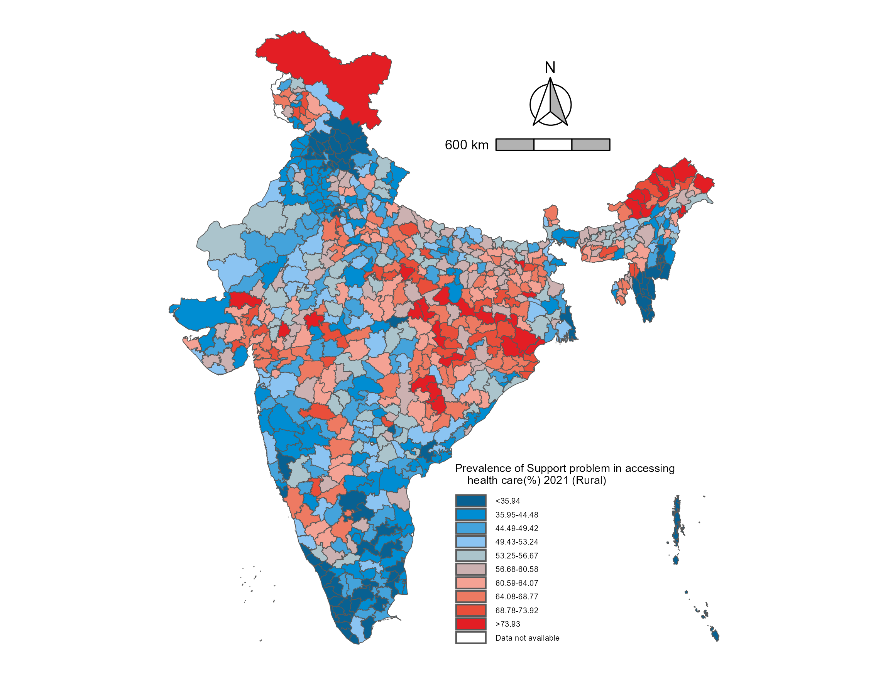 | 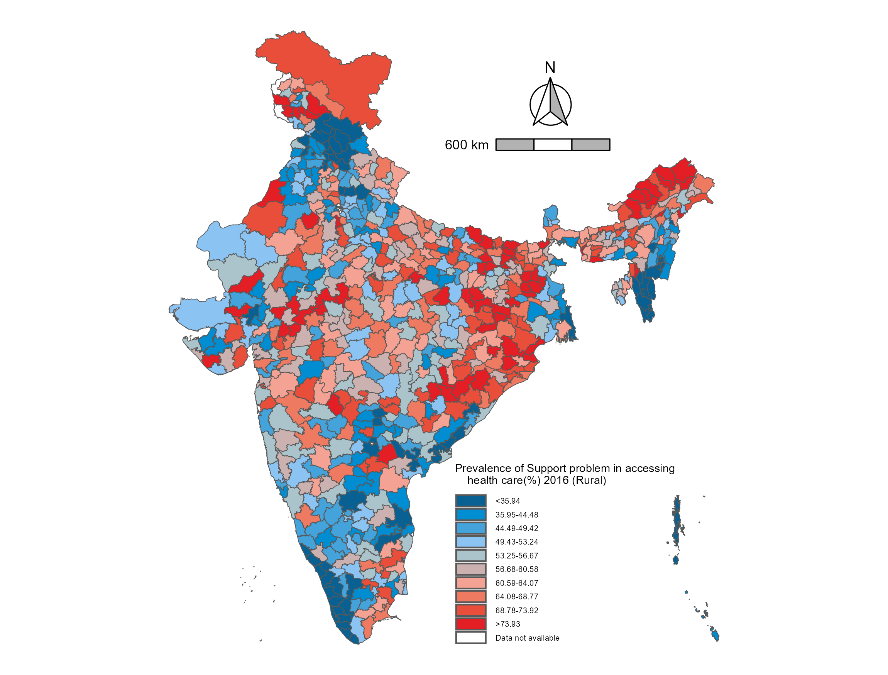 | 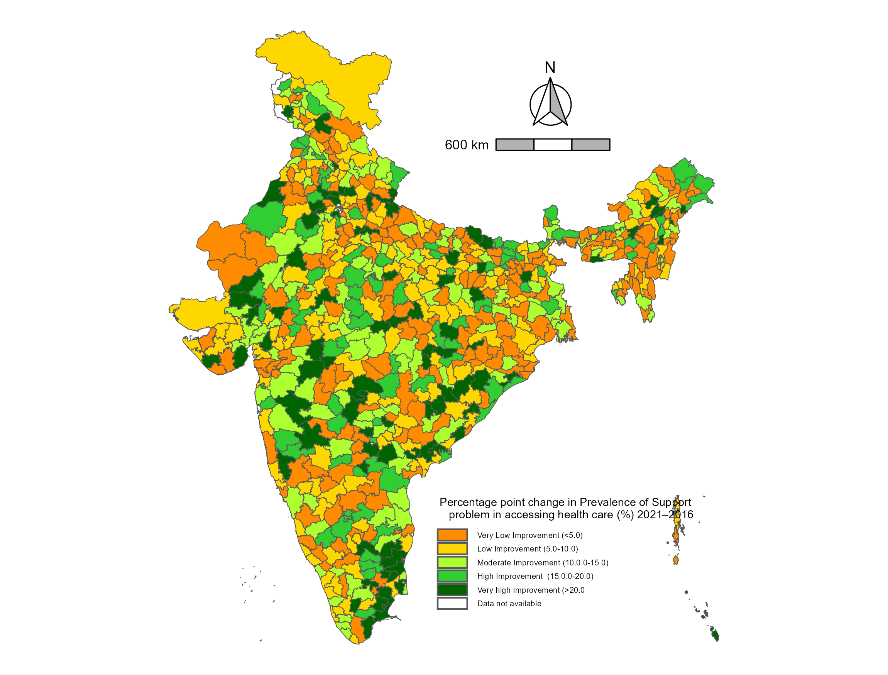 |
